# Doctors' Fees and Patients' Payments

**Published:** 1921-08-06

**Authors:** 


### The Hospital

The Workers' Newspaper of Administrative Medicine and Institutional Life, Administration, National Insurance and Health.

No. 1835, Vol. LXX. SATURDAY, AUGUST 6, 1921. PRICE TWOPENCE

### DOCTORS' FEES AND PATIENTS' PAYMENTS.

From the hospital point of view, with the exception of two important resolutions, the annual meeting of the British Medical Association at Newcastle presented no outstanding feature. The assembly expressed its belief in the voluntary system, and defined the position of medical practitioners with regard to the extension of the uses of Poor-law infirmaries, to contributing patients, and to provident schemes. But when this representative meeting discussed the Cave Report and the matter of patients' payments in voluntary hospitals, two decisions were arrived at which will undoubtedly be received by the hospitals in the first place with surprise, and secondly with opposition.

It will be remembered that the Cave Committee took the common-sense and generally accepted view that the time-honoured bargain between honorary staffs and hospitals is beneficial to both the institution and the honoraries. But such benefits as accrue to the visiting staffs are indirect, and consequently their service has always been regarded by themselves and others as voluntary. Clearly this is so, because the honoraries have always claimed the right to join on equal terms with the voluntary members of the Boards and Committees in managing the institutions—a privilege which has never been accorded to pharmacists, matrons, and others, and clearly marks the difference between paid and unpaid workers. But the Newcastle meeting " maintains that the essence of the voluntary hospital system is the independent and voluntary management, and that this is in no way related to the conditions of service of the medical staff." The doctors cannot have it both ways, and if a logical effect is to be given to this resolution the constitution of many hospitals, as defined in Acts of Parliament, Royal Charters, Board of Trade articles, or however expressed, must be revised in order that the medical staffs shall no longer join in the deliberations of their lay associates. This will be a revolution which, at a difficult time, will take from the hospitals an advantage which was not gained in past times without a struggle on the part of the honorary medical staffs, who at long last have succeeded in convincing the lay workers of its efficacy and value.

The foregoing resolution was passed without opposition. This cannot be said of the resolution with regard to payments by patients. It was sought to insist on twenty per cent. of all patients' payments being passed to a fund at the disposal of the honorary staff. This, to put it rather crudely, means in one of its aspects that if a patient, wishing to show his gratitude, or his wish to be as independent as his position allows, agrees to give half-a-crown a week to the hospital, while he is an inmate, to help meet the cost of the food he consumes or of the general standing charges that have to be met on his behalf, sixpence of this modest sum is to be handed to the honorary medical staff to distribute among their body, or otherwise to use as they think fit. Little wonder that such a paltry suggestion met with substantial opposition. One member said that anything of the kind would bring the honorary medical staffs into disrepute with the public; he instanced one well-known institution in the North of England where ninety per cent. of the income is obtained from such contributions, and where a twenty per cent. deduction would land the institution in perpetual debt. In the result the resolution, amended to leave the amount of deduction undefined, was carried by only a two-thirds majority, the division probably revealing a cleavage between the general practitioner and the honorary staffs. As Lord Cave's report said, " the honorary staffs of some hospitals are unwilling to share in such a fund; and two distinguished physicians expressed the view that if the medical staffs came to be subsidised to any substantial extent ' the bottom would drop out of the voluntary system.' "

We have every sympathy with the medical men who may desire to uphold their rights in the changing conditions of organised healing in this country. That they should step warily, see clearly, and speak unequivocally when their patients are being admitted to rate and voluntarily supported institutions, under conditions which have never prevailed before, is so understandable that it should secure public sympathy. That the institutions should not profit at the expense of the profession by receiving patients at a charge which exceeds the cost of maintenance; and that patients, V.D. cases, school children, insurance cases, pensioners, and others for whom the State has made itself responsible, should not be received while the interests of the practitioner and consultant are disregarded, are principles readily conceded. But there is a wide distinction between such payments and those of the quasi-donation type, which are in principle indistinguishable from Hospital Saturday contributions, workmen's contributions, and patients' valedictory gifts of gratitude. One-third of the members of the Newcastle meeting were aware of this, and two-thirds were not. We feel that if this regrettable decision is pressed a further and profound embarrassment will be thrust upon the voluntary hospital system, at a most inopportune time, by those who have always been counted amongst its staunchest supporters.

